# A Polarizer-Free Electro-Optical Switch Using Dye-Doped Liquid Crystal Gels

**DOI:** 10.3390/ma2041662

**Published:** 2009-10-26

**Authors:** Yi-Hsin Lin, Hung-Chun Lin, Jhih-Ming Yang

**Affiliations:** Department of Photonics, National Chiao Tung University 1001 Ta Hsueh Rd., Hsinchu 30050, Taiwan

**Keywords:** liquid crystal, polarizer-free, electro-optical switch, LC gels

## Abstract

We demonstrate a polarizer-free electro-optical switch using dye-doped liquid crystal (LC) gels. The mechanism of dye-doped LC gels mainly involves the combination of polymer scattering and dye absorption. However, the domain size of polymer networks, dye concentration, LC concentration, and fabrication process can all affect the phase separation process and thus result in dye-doped LC gels with different electro-optical performance. We have studied experimentally the factors which can affect the dye-doped LC gels. The potential applications for dye-doped LC gels are flexible displays and electrically tunable light shutters.

## 1. Introduction

Most electro-optical switches using liquid crystals (LC) require at least one polarizer [1,2,3]. These polarizers limit the optical efficiency and viewing angle. In order to remove the polarization dependency of LC-based electro-optical switches, absorption-based Guest-Host systems or dye-doped LC systems which have built-in crossed polarizers are proposed, such as Cole-Kashnow cells, White-Taylor cells, or double orthogonal cells [2,3]. Besides light absorption, dye-doped polymer-dispersed liquid crystals (dye-doped PDLC) consisting of randomly dispersed dye-doped LC droplets are still polarizer-free by combining the extra effect of scattering [4,5]. However, the electro-optical performance of dye-doped PDLCs is limited due to the dye molecules entangled in the polymer matrix, the order parameter of the dichroic dye and the dichroic ratio (typically ~10:1) of the dye. To avoid the problem of solubility between dye and polymer matrix, we have developed a polarizer-free electro-optical switch using a dye-doped dual-frequency liquid crystal (DFLC) gel and dye-doped LC gels [6,7,8,9]. Without polarizers, the optical efficiency is high and the viewing angle is wide. The gel-like features of dye-doped LC gels, vertically aligned polymer networks and low temperature processes are suitable for trimable and bendable polarizer-free flexible displays in reflective mode. However, the factors which can affect the performance of dye-doped LC gels have not been reported yet.

In this paper, several factors affecting the electro-optical properties of dye-doped LC gels such as curing temperature, UV curing intensity, monomer concentration, and cell gap are discussed experimentally. Moreover, the optical analysis is also discussed. Some potential applications of dye-doped LC gels are flexible displays and electrically tunable light shutters.

## 2. Sample Preparation and Operating Principle

**Figure 1:**
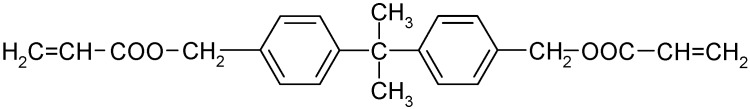
Structure of the diacrylate monomer.

The dye-doped LC mixture consists of a ZLI-4788 negative nematic liquid crystal (Merck, n_e_ = 1.6567, Δn = 0.1647 at λ = 589 nm; Δε = −5.7 at f = 1 kHz) and a M1 diacrylate monomer (bisphenol-A-dimethacrylate) and a dichroic dye S428 (Mitsui, Japan) at 90:5:5 wt% ratios. The structure of the diacrylate monomer is as shown in Figure 1.

**Figure 2:**
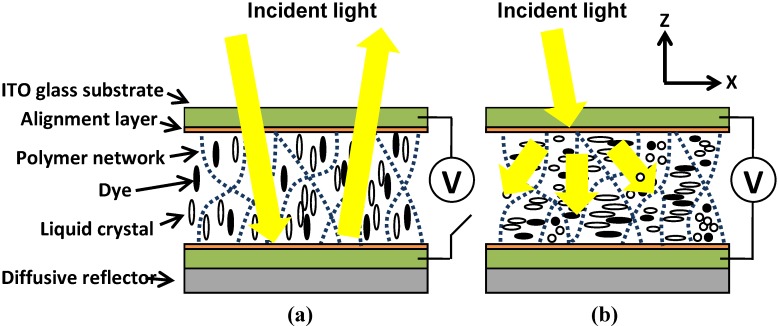
Schematic operating principle of dye-doped gels at (a) voltage-off state and (b) voltage-on state.

We then injected the mixture into an empty cell consisting of two glass substrates coated with a conductive layer of indium-tin-oxide (ITO) and a layer of polyimide (PI) without a rubbing treatment which is a mechanical process to create micro-grooves along the rubbing direction. The thicknesses of both layers are around 100 nm. The PI layer provides the vertical alignment for LC directors. The cell gap, the distance between two substrates, was 5 μm. The filled cell of the dye-doped LC mixture was then exposed to UV light (λ~365 nm, I~ 2.6 mW/cm^2^) at a fixed temperature (10 °C) for 90 minutes. This fixed temperature for UV curing process is called the curing temperature. After photo-polymerization, chain-like polymer networks are formed along the z direction because LC directors are aligned along this direction by the vertical alignment layer, as shown in Figure 2(a).

Figure 2 shows the operating principles of dye-doped LC gels. In the absence of an applied voltage polymer networks, dichroic dyes, and liquid crystal directors are perpendicular to the glass substrates. The ordinary refractive index of the liquid crystals matches the refractive index of the polymer networks, so the cell has less scattering and weak absorption. The cell is in the bright state. When a voltage with a frequency of 1 kHz larger than the threshold voltage is applied, the negative liquid crystals and dye molecules tend to reorient perpendicular to the z direction with the same tilt angle but random orientations owning to the no mechanically rubbing treatment in the PI layers. [10] The cell then switches into multi-domain mode. The scattering and absorption increase. It is polarization independent. When we apply a larger voltage (V), the LC directors and dye molecules are randomly distributed along the x-y plane, as shown in Figure 2(b). Both scattering and absorption are strong and polarization independent for two reasons. One is the mismatch between the refractive index of polymer networks and the extraordinary refractive index of liquid crystals. The other is all the dye molecules are along the x-y plane; as a result, all polarization of incoming light experience the same averaged absorption effect. By controlling the driving voltage, dye-doped LC gel can be a polarizer-free electro-optical switch.

**Figure 3:**
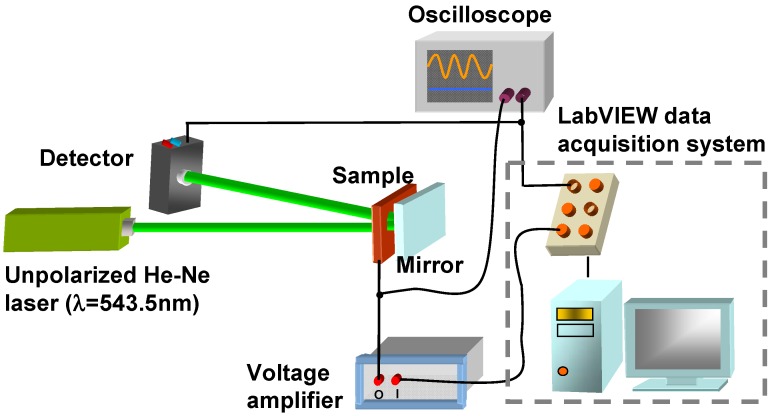
Experimental setup for the reflectance measurements.

## 3. Experimental Section

In order to measure the electro-optical properties of dye-doped LC gels, we adopted the typical reflectance measurement setup shown in Figure 3. We used an unpolarized green He-Ne laser (λ = 543.5 nm, Melles Griot, Model 05-LGR-173) as the incident light source. The light double passes through the cell by putting a dielectric-reflected mirror behind the sample and the corresponding collection angle is around 5°. We measured the reflectance with a large area photodiode detector (New Focus, Model 2031) which was placed ~23 cm behind the sample. The distance between the detector and the sample is larger; the measuring reflectance is smaller because of the scattering effect. The reflectance is normalized to that of a pure LC cell with the same cell gaps. The Labview data acquisition system was used to applied voltages and collect light intensity at the same time. The response time was measured by an oscilloscope. In the following sections four effects are discussed including the effects of the curing temperature, the UV curing intensity, the monomer concentration, and the cell gap.

### 3.1. Effect of Curing Temperature

**Figure 4:**
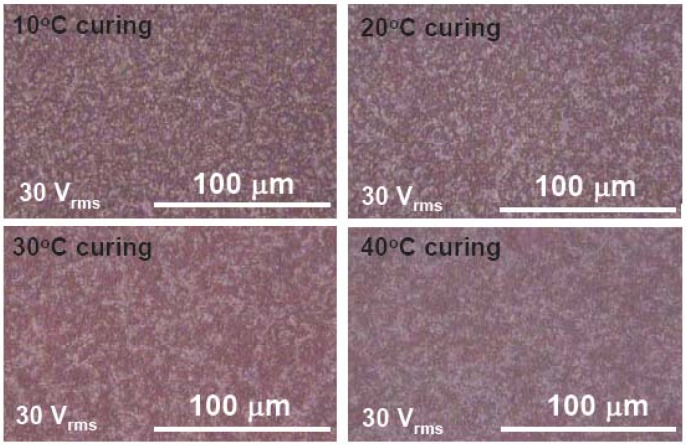
The microscopic images of dye-doped LC gels with various curing temperature. The applied voltage is 30 V_rms_.

We summarize the effect of curing temperature in this section since we have previously discussed this topic [8,11]. Figure 4 shows the morphologies observed under a microscope at 30 V_rms_ and different curing temperatures.

At V = 0, the cell shows a bright state because of the vertically aligned polymer networks, LC and dye molecules. At 30 V_rms_, it shows the fine domain textures of the polymer networks, and appears red because of dye molecules, as shown in Figure 4. The higher curing temperature, the larger the domain size of dye-doped LC gels. The domain size affects the electro-optical properties of dye-doped LC gels. The LC molecules are easily rotated by the applied voltage as a weak anchoring energy; therefore, dye-doped LC gels have a lower threshold voltage and a slower decay time with larger domain sizes. The threshold voltage is the voltage at which the reflectance starts to decrease. The threshold voltage decreases from 5.82 (10 °C) to 3.72 V_rms_ (40 °C) and decay time increases from 6 ms (10 °C) to 22.7 ms (40 °C). The reflectance at V=0 decreases from 55% (10 °C) to 42% (40 °C) with the increases of the curing temperature. That is because the better vertical alignment of LC directors, dye molecules and polymer networks at smaller domain size. The raising times are all around 0.4 ms at different curing temperature. The contrast ratio, a reflectance ratio of 0 V to 30 V_rms_, is ~450:1 at 10 °C, 250:1 at 20 °C, 200:1 at 30 °C, and 300:1 at 40 °C. The contrast ratio decreases at T < 30 °C due to the larger polydomain and then increases as T > 30 °C caused by the dynamic scattering, a fluctuation of liquid crystal directors in polymer domains, which help decreasing the dark state reflectance. The reason why the larger domain has the dynamic scattering is still unclear.

**Figure 5:**
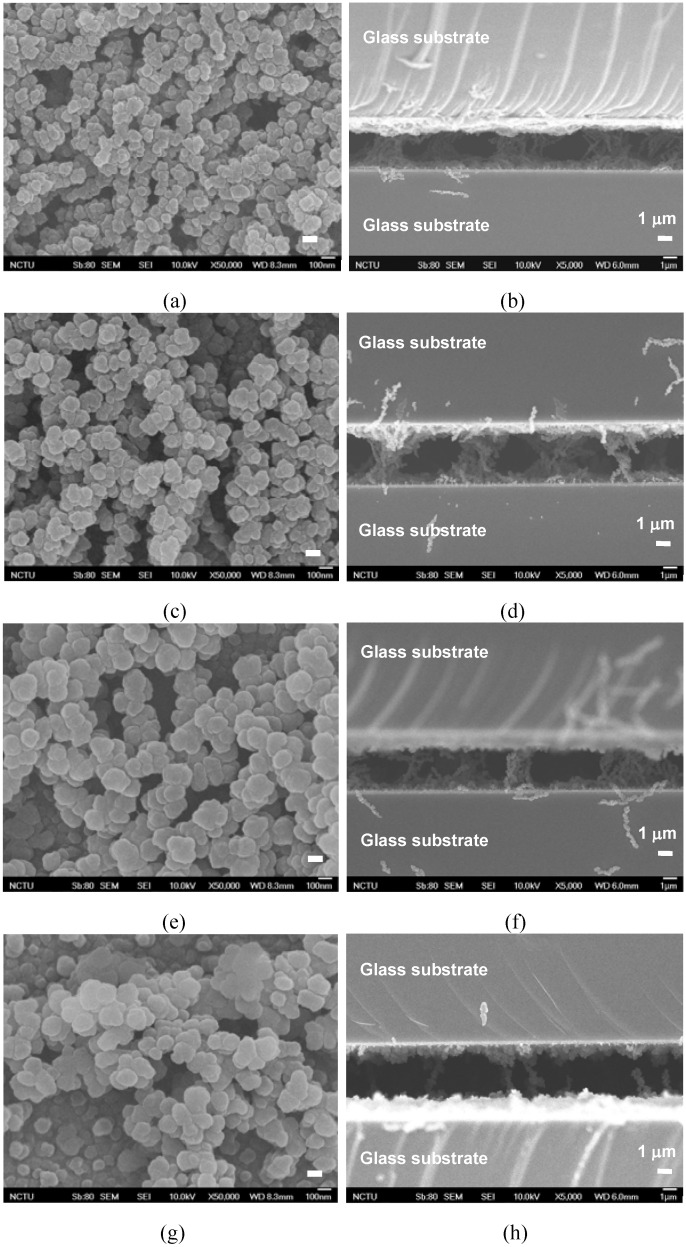
SEM photographs of dye-doped LC gels at curing temperatures 10 °C (a, b), 20 °C (c, d), 30 °C (e, f), and 40 °C (g, h). The LC and dye were extracted. (a), (c), (e) and (g) are the top views of the cells. (b), (d), (f), and (h) are the side views of the cells. The white-indicated bars in (a), (c), (e) and (g) are 100 nm.

Scanning Electron Microscopy (SEM) photographs after removing LC and dye molecules with hexane are shown in Figure 5(a)-(h). Figure 5(a), Figure 5(c), Figure 5(e) and Figure 5(g) are the top views of the cells at different curing temperatures (T). Figure 5(b), Figure 5(d), Figure 5(f), and (Figure 5h) are the side views of the cells at different curing temperatures. In Figure 5(b), Figure 5(d), Figure 5(f), and Figure 5(h), the polymer networks are perpendicular to the glass substrates. The polymer networks of dye-doped LC gels consist of chain-linked polymer grains. The averaged sizes of polymer grains measured from Figure 5(a), Figure 5(c), Figure 5(e) and Figure 5(g) are around 68 nm at T = 10 °C, 94 nm at T = 20 °C, 125 nm at T = 30 °C, and 132 nm at T = 40 °C. The averaged domain sizes of polymer networks measured from Figure 5(b), Figure 5(d), Figure 5(f), and Figure 5(h) are around 3.25 μm at T = 10 °C, 4.62 μm at T = 20 °C, 4.78 μm at T = 30 °C, 6.12 μm at T = 40 °C. Both of the domain sizes and the size of polymer grains increase with curing temperatures. The scattering is mainly because of the domain sizes of polymer networks are near wavelength of incident light while the sizes of polymer grains are smaller than the wavelength.

### 3.2. Effect of UV Curing Intensity

**Figure 6:**
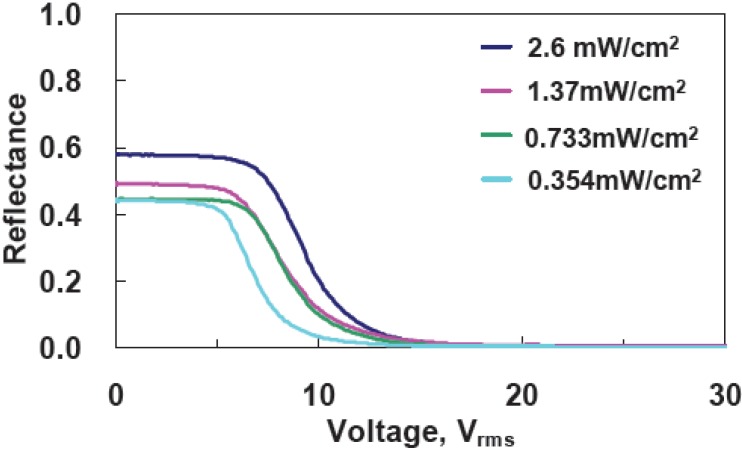
Voltage-dependent reflectance at various curing intensities at a curing temperature of 10 °C.

**Figure 7:**
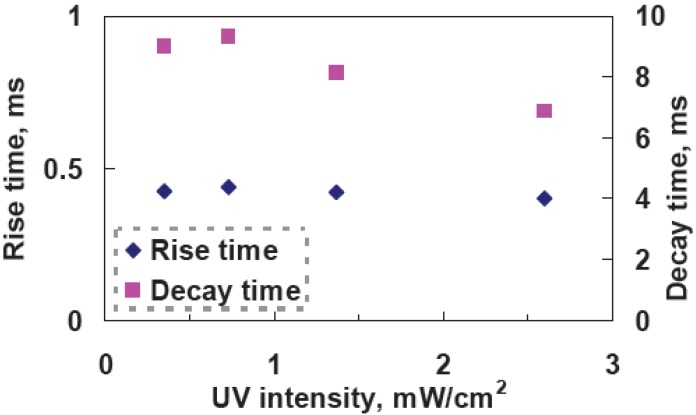
Measured response time as a function of UV curing intensity at a curing temperature of 10 °C.

To examine the effect of UV curing intensity (I), we prepared four samples with the same mixtures at the curing temperature 10 °C, but at different UV curing intensities, which were 2.6, 1.37, 0.733, 0.354 mW/cm^2^. The cell gaps were 5 μm. The reflectance as a function of a voltage is shown in Figure 6. With the increases of UV curing intensity, threshold voltage (V_th_) increases from 4.82 V_rms_ (I ~ 0.354 mW/cm^2^) to 5.92 V_rms_ (I~2.6 mW/cm^2^ ). The reflectance at V = 0 increases from 44% (I ~ 0.354 mW/cm^2^ ) to ~57% ( I~2.6 mW/cm^2^). In Figure 7, rise time is around 0.4 ms, but decay time decreases from 9 ms (I ~ 0.354 mW/cm^2^) to 6.88 ms (I ~ 2.6 mW/cm^2^). The larger UV curing intensity results in smaller domain size of polymer networks. That causes the stronger anchoring energy and then enlarges the threshold voltage and boost the response time. The reflectance at V = 0 decreases under higher UV curing intensity owing to better vertically alignment at V = 0.

### 3.3. Effect of the Monomer Concentration

**Figure 8:**
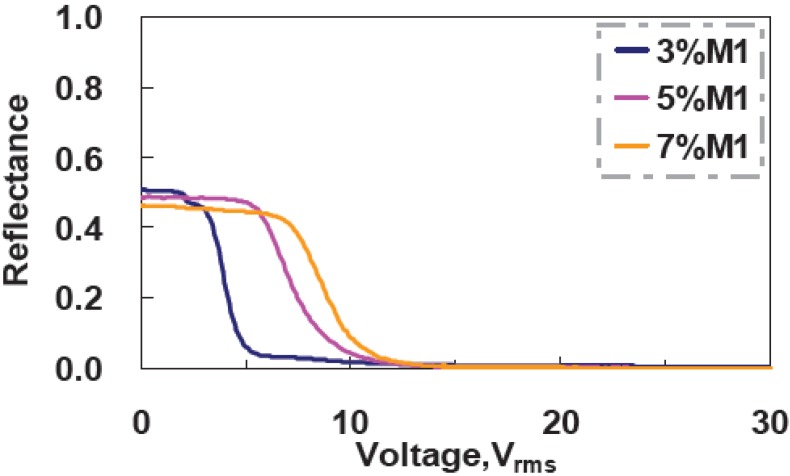
Voltage-dependent reflectance at three different monomer concentrations.

In this section, the monomer concentration effect is discussed. We prepared three samples with different monomer concentrations of 3 wt%, 5 wt%, and 7 wt%. The fabrication processes of the samples were the same at the UV curing intensity 2.6 mW/cm^2^ at the curing temperature 20 °C. The cell gaps were still 5 μm. The voltage-dependent reflectance at different monomer concentrations is shown in Figure 8.

### 3.4. Cell Gap Effect

**Figure 9:**
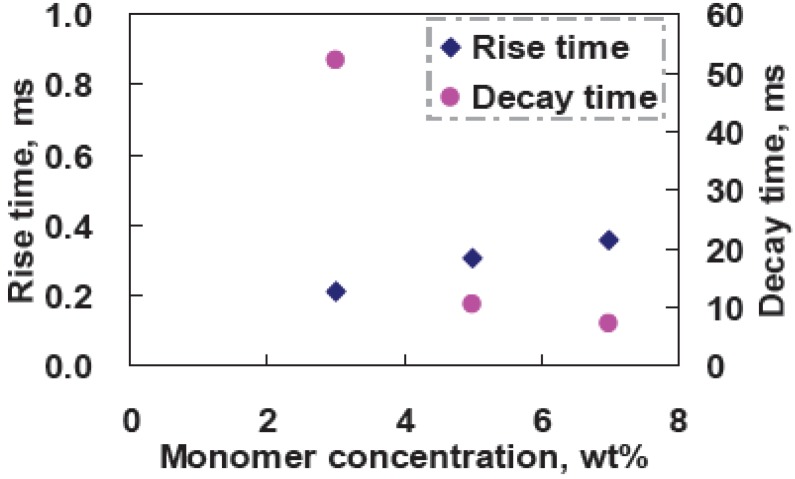
Measured response time as a function of monomer concentration.

The threshold voltage increases from 2.1 V_rms_ (at 3 wt% M1) to 6.52 V_rms_ (at 7 wt% M1) with the monomer concentration due to the denser polymer networks. The reflectance at V = 0 decrease slightly (from 51% at 3 wt% M1 to 46% at 7 wt% M1). That is because denser polymer networks affect the vertical alignment of LC directors and also increase the scattering. CR increases from 222:1 (at 3 wt% M1) to 486:1 (at 7 wt% M1) owning to better scattering of higher monomer concentration at the high driving voltage. Figure 9 shows the measured response time as a function of UV curing intensity. Rise time is around 0.2 ms-0.4 ms, and decay time increases from 52 ms (at 3 wt% M1) to 7.3 ms (at 7 wt%M1). The higher monomer concentration has higher anchoring energy of polymer networks; therefore, the LC directors are relaxed back faster after turning off the applied voltage [12].

**Figure 10:**
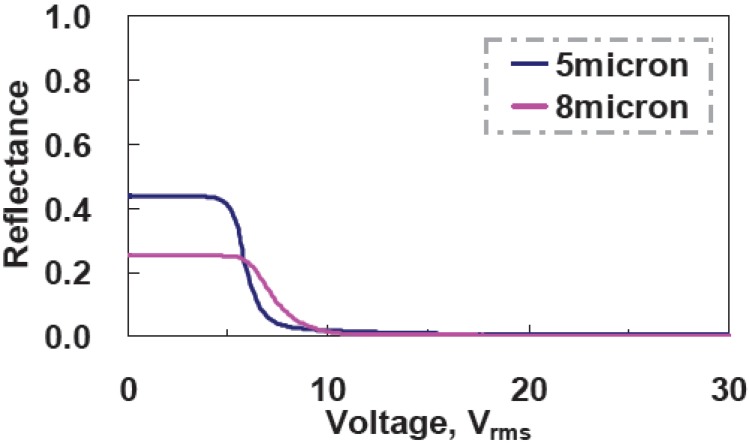
Voltage-dependent reflectance of different cell gaps.

We can also increase the cell gap in order to enlarge the scattering and absorption of incident light. Two samples with a mixture of ZLI4788, M1, and S428 at 90:5:5 wt% ratios are made under the same curing condition:curing temperature ~30 °C and UV curing intensity ~2.6 mW/cm^2^. The cell gaps of two samples are 5 μm and 8 μm. As one can see in Figure 10, the reflectance at V=0 drops from 44% for 5 μm cell gap to 25% for an 8 μm cell gap, even though CR increases from 208:1 to 264:1 due to the better dark state in the larger cell gap. The large cell gap can help scattering and absorption; however, the trade off is low bright sate, the reflectance at V=0. Therefore, it is not worth increasing the cell gap.

### 3.5. Performance of Dye-Doped LC Gels

**Figure 11:**
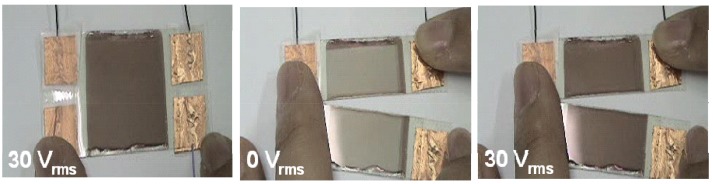
The performance of dye-doped LC gels. Left: before trimming at 30 V_rms_. Middle: after trimming at 0 V_rms_. Right: after trimming at 30 V_rms_.

**Figure 12:**
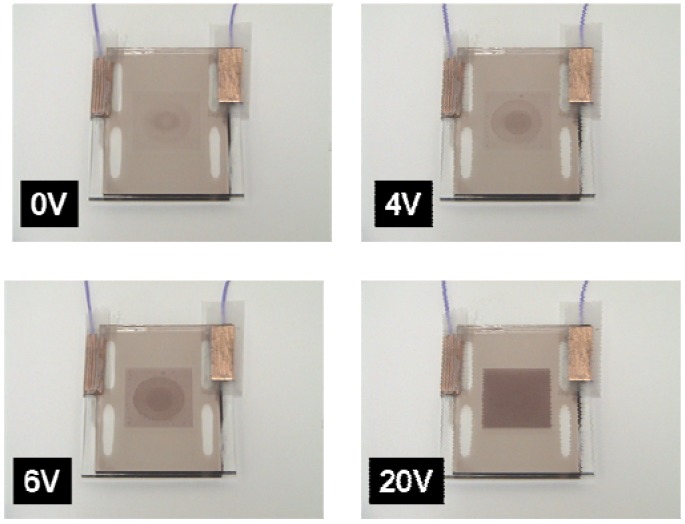
The performance of electrically tunable band-pass filter using dye-doped LC gels at 0, 4, 6 and 30 V_rms_. Middle: after trimming at 0 V_rms_. Right: after trimming at 30 V_rms_.

The images of a single pixel of the flexible polarizer-free electro-optical switch using dye-doped LC gels at V = 0 and V = 30 V_rms_ are shown in Figure 11. A piece of white paper was used as a diffusive reflector. The ambient white light was used to illuminate the sample. By replacing glass substrates with flexible substrate, dye-doped LC gel is not only bendable but also trim-able because our material is gel-like. The flexible substrates are provided by EOL/ITRI (Electronics& Optoelectronics Research Laboratories, Industrial Technology Research Institute, Taiwan). IZO was over coated on the top of flexible substrates made by polycarbonate with thickness 120 μm. The cross shaped microstructures made by photo-spacers, resins, were developed on the flexible substrates by photolithography process. The width of photo-spacers is 10 μm and the pitch of photo-spacers is 430 μm. The flexible dye-doped LC gel remains similar performance after cutting by a scissor. In Figure 12, we fabricated an electrically tunable band-pass filter by a spatial distribution of polymer network densities of dye-doped LC gels.

## 4. Discussion

By considering the scattering and absorption, the reflectance (R(θ)) as a function of tilt angle (θ) of LC directors with respect to x-axis can be expressed as:

(1) $$R(\theta )\approx e^{-\alpha_{ave}(\theta )\cdot 2d}\cdot e^{-\beta_{ave}(\theta )\cdot 2d}$$

where d is cell gap, α_ave_(θ) is the average absorption coefficient, and β_ave_(θ) is the average scattering coefficient. α_ave_(θ) and β_ave_(θ) satisfy the following equations:

(2) $$\alpha_{ave}(\theta )=\rho_{1}\cdot \frac{\alpha_{eff}(\theta )+\alpha_{⊥}}{2}$$

(3) $$\beta_{ave}(\theta )=\rho_{0}\cdot \frac{\sigma_{eff}(\theta )}{V}$$

where ρ_1_ is the dye concentration, α_//_ and α_⊥_ are the absorption coefficients when the polarization of incident light is parallel or perpendicular to the principal axis of dye molecule. ρ_0_ is the LC concentration, V is the average volume of a droplet. α_eff_(θ) can be expressed as:

(4) $$\alpha_{eff}(\theta )=\frac{\alpha_{//}\cdot \alpha_{⊥}}{\sqrt{\alpha_{//}\cdot {\text{sin}}^{2}\theta +\alpha_{⊥}\cdot {\text{cos}}^{2}\theta }}$$

We can estimate α_//_ = 11.83 μm^-1^ and α_⊥_ = 0.962 μm^-1^ based on the experimental results. σ_eff_ in Equation (3) is the effective scattering cross section from all liquid crystal droplets and can be expressed as:

(5) $$\sigma_{eff}(\theta )=\frac{1}{\pi }\int_{0}^{\pi }\sigma_{s}\left(\theta ,\alpha_{o}\right)\cdot d\alpha_{o}$$

Based on anomalous diffraction approach [4,13], scattering cross section results from a single LC droplet is;

(6) $$\sigma_{s}\left(\theta ,\delta \right)=2\sigma_{o}\left[H_{ve}(\theta )\cdot Cos^{2}\delta +H_{vo}\cdot Sin^{2}\delta \right]$$

where σ_0_ is the geometrical optics cross section related to the domain size, δ is the polarization angle; H_ve_(θ) and H_vo_(θ) stand for phase shift induced by e-ray and o-ray respectively. The averaged domain sizes of polymer networks are measured around 1.5 μm~3 μm.

**Figure 13:**
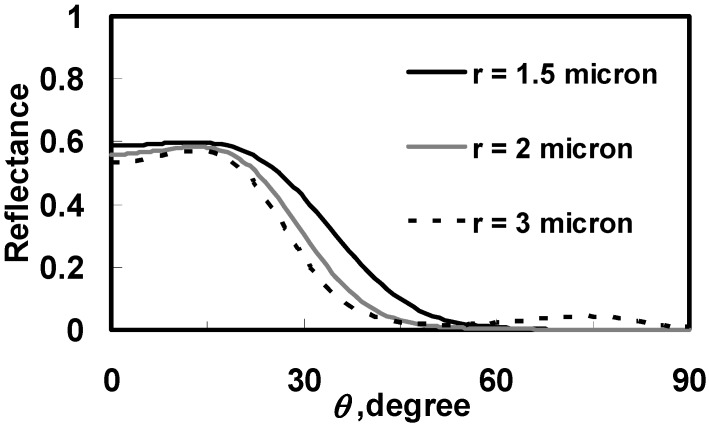
Calculated reflectance as a function of tilt angle in dye-doped LC gels at different domain sizes.

For numerical calculations we have chosen the following parameters according to the experiments: ρ_1_ = 0.05 g/cm^3^, and ρ_0_ = 0.89 g/cm^3^. α_//_ = 11.83 μm^-1^ and α_⊥_ = 0.926 μm^-1^. The simulation result is shown in Figure 13. In Figure 13, the reflectance decreases with the tilt angle. The R-θ curve shifts to right as the domain size is smaller. The simulation results agree with the experimental results [8]. The smaller domain size or larger density of polymer networks can result in the larger operating voltage and better dark state. We can adjust UV intensity, curing temperature, the controlled temperature under UV illumination, and concentration of LC, dye or monomer to change the domain sizes of polymer networks.

## 5. Conclusions

We have investigated the factors affecting the electro-optical properties of a polarizer-free electro-optical switch, the dye-doped liquid crystal gel. The mechanism of dye-doped LC gels mainly involves a combination of scattering and light absorption. The absorption results from the dichroic dye molecules. The scattering comes from the polymer network domains. The low curing temperature, strong UV curing intensity, and more monomer concentration result in good CR and fast response time because of large scattering caused by small domains. However, small domains cause large anchoring energy in liquid crystals. The driving voltage is then increased. Enlarging the cell gap to elongate the optical path of scattering and absorption is a way to improve the dark state at 30 V_rms_. The tradeoff is the bright state at 0 V_rms_. The optical analysis agrees with the experimental results. The potential applications of dye-doped LC gels include flexible displays, electrically tunable iris, and electrically tunable band-pass filters, and electrically tunable light shutters.
